# Biological Responses to Perfluorododecanoic Acid Exposure in Rat Kidneys as Determined by Integrated Proteomic and Metabonomic Studies

**DOI:** 10.1371/journal.pone.0020862

**Published:** 2011-06-03

**Authors:** Hongxia Zhang, Lina Ding, Xuemei Fang, Zhimin Shi, Yating Zhang, Hebing Chen, Xianzhong Yan, Jiayin Dai

**Affiliations:** 1 Key Laboratory of Animal Ecology and Conservation Biology, Institute of Zoology, Chinese Academy of Sciences, Beijing, People's Republic of China; 2 National Center of Biomedical Analysis, Beijing, People's Republic of China; INSERM, France

## Abstract

**Background:**

Perfluorododecanoic acid (PFDoA) is a perfluorinated carboxylic chemical (PFC) that has broad applications and distribution in the environment. While many studies have focused on hepatotoxicity, immunotoxicity, and reproductive toxicity of PFCAs, few have investigated renal toxicity.

**Methodology/Principal Findings:**

Here, we used comparative proteomic and metabonomic technologies to provide a global perspective on renal response to PFDoA. Male rats were exposed to 0, 0.05, 0.2, and 0.5 mg/kg/day of PFDoA for 110 days. After 2-D DIGE and MALDI TOF/TOF analysis, 79 differentially expressed proteins between the control and the PFDoA treated rats (0.2 and 0.5 mg-dosed groups) were successfully identified. These proteins were mainly involved in amino acid metabolism, the tricarboxylic acid cycle, gluconeogenesis, glycolysis, electron transport, and stress response. Nuclear magnetic resonance-based metabonomic analysis showed an increase in pyruvate, lactate, acetate, choline, and a variety of amino acids in the highest dose group. Furthermore, the profiles of free amino acids in the PFDoA treated groups were investigated quantitatively by high-coverage quantitative iTRAQ-LC MS/MS, which showed levels of sarcosine, asparagine, histidine, 1-methylhistidine, Ile, Leu, Val, Trp, Tyr, Phe, Cys, and Met increased markedly in the 0.5 mg dosed group, while homocitrulline, α-aminoadipic acid, β-alanine, and cystathionine decreased.

**Conclusion/Significance:**

These observations provide evidence that disorders in glucose and amino acid metabolism may contribute to PFDoA nephrotoxicity. Additionally, α_2u_ globulin may play an important role in protecting the kidneys from PFDoA toxicity.

## Introduction

Perfluorinated carboxylic chemicals (PFCs) have been manufactured and used in various industrial and commercial products over the past 50 years, including surfactants, lubricants, fire fighting foams, and cosmetics [1]. Their high-energy carbon-fluorine bonds enable them to resist hydrolysis, photolysis, biodegradation, and metabolism, which increases their impact on environmental and human health [2]. Recent biomonitoring studies have revealed significant global distribution of PFCs in the environment, wildlife, human beings [1], [3]–[6], and even remote areas such as the Arctic [7], [8]. Perfluorooctanoic acid (PFOA, C8), perfluorooctansulfonate (PFOS, C8), and perfluorododecanoic acid (PFDoA, C12) are the most commonly detected PFCs [9] and have thus received much attention from toxicologists and environmental scientists.

The liver and kidney are the main organs for PFCs bioaccumulation in animals [10]. In addition, the liver is the primary target organ for PFCs toxicity [11], [12], and the kidney is the main elimination organ for PFCs [13]. Studies have shown that exposure to PFCs increases the liver-to-body weight ratio, hepatocellular hypertrophy, and peroxisome proliferation [11], [12], [14] and induces adenoma in hepatocytes in laboratory animals [15], [16]; however, few studies have focused on the toxic effects of PFCs on the kidney. While Kawashima *et al*. [17] reported that PFOA induces peroxisomal β-oxidation in rat kidney, Son *et al*. [18] observed no renal toxicity in mice orally exposed to PFOA for 21 days. Our previous study found that several kidney damage biomarkers (specifically, blood urea nitrogen (BUN), creatinine, and BUN to creatinine ratio) increased significantly in rat serum after chronic exposure to PFDoA for 110 days compared with the control group [19], which compelled our further investigation of the potential renal toxicity of PFCs.

Two-dimensional difference gel electrophoresis (2-D DIGE) is an efficient and accurate method for the separation of proteins in complex mixtures and quantifies differential expression in treated and control samples [20]. This technology is an effective broad-based screening tool for the analysis of environmental stress responses in organisms [21]. Metabonomics is a systems biology approach for determining the endogenous metabolic responses of living systems using either ^1^H nuclear magnetic resonance (NMR) spectroscopy or mass spectrometry in conjunction with statistical pattern recognition [22].

We used 2-D DIGE followed by mass spectrometric analyses of individual protein spots to better understand the potential renal toxicity of PFDoA. NMR-based metabonomic analysis was employed for lipid and aqueous kidney extracts to determine the varied metabolite profiles from normal and PFDoA treated rats. In addition, to accurately detect variations in the profiles of free amino acids in kidneys exposed to PFDoA, we used iTRAQ®–LC–MS/MS with 42 internal standards of physiological amino acids and amines for absolute quantification by isotope ratio analysis. These data will help estimate an organism's internal reserves and the degree of metabolic disintegration under the influence of xenobiotics and adaptive abilities of the target organs and the organism as a whole. This research will not only explore the underlying mechanisms of PFDoA nephrotoxicity but will also provide a reference for assessing the risk of PFCs to human health.

## Materials and Methods

### Ethics Statement

This study was conducted in accordance with the Animal Ethics Committee of Institute of Zoology, Chinese Academy of Sciences. The institute does not issue a number or ID to any animal study, but the ethical committee guides the animal use.

### Experiment animals and design

Male Sprague-Dawley rats (230–240 g) were obtained from the Weitong Lihua Experimentary Animal Central (Beijing, China). Rats were housed in a light-controlled room under a 12-h light/dark cycle with *ad libitum* access to food and water. The ambient temperature in the animal room was 20–26°C and the relative humidity was 40–60% under the care of the Laboratory Animal Unit, Institute of Zoology, Chinese Academy of Sciences. After one week of adaptation, the rats were separated into four groups of ten animals. The PFDoA (CAS No. 307-55-1, 95% purity, Sigma-Aldrich (St. Louis, MO)) was dissolved in 0.2% Tween-20. The treatment rats were given doses of 0.05, 0.2, and 0.5 mg PFDoA/kg body weight/day by oral gavage for 110 days. The control animals were treated with 0.2% Tween-20 (vehicle) alone. At the end of the experiment, six rats were selected randomly from each group and were weighed and euthanized by decapitation. The remaining four rats from each group were used for another study. The left kidney of each euthanized rat was removed immediately, washed with PBS, weighed, divided into four small aliquots, flash frozen in liquid nitrogen, and stored at −80°C for further analysis. Samples for the same analysis were from the same region of the kidney.

### Protein preparation and CyDye labeling

Total protein was extracted from the kidney using sample lysis buffer (7 M urea, 2 M Thiourea, 30 mM Tris, 4% (w/v) CHAPS, 1 mM PMSF and 1% protease inhibitor cocktail (Sigma-Aldrich, St. Louis, MO). Protein concentration was determined using a 2-D Quant protein assay kit (GE Healthcare, Uppsala, Sweden).

We chose three groups (0, 0.2, and 0.5 mg/kg/day PFDoA) for DIGE analysis based on previously observed gene expression changes in the liver and clinical chemistry parameters [19]. Equal amounts of protein sample from two randomly selected rats from the same treatment group were pooled and purified using a 2-D Clean-up kit (GE Healthcare) for subsequent DIGE analysis. Each group yielded three pooled protein samples, and the pH values of the desalted samples were adjusted to 8.5 with 100 mM sodium hydroxide before labeling. Proteins were labeled with CyDye Fluor minimal dyes (GE Healthcare) according to the manufacturer's recommended protocols. The internal standard (IS) was comprised of a pooled equal amount from all experimental samples. A total of 50 µg of protein from the treated and control groups were labeled with 400 pmol of either Cy3, Cy5, or Cy2 (Cy2 was used to label the IS). The labeled mixtures were combined according to Table S1 and were then adjusted to 450 µl with rehydration buffer (7 M urea, 2 M Thiourea, 2% CHAPS, 0.5% IPG buffer pH 4–7, and a trace of bromophenol blue) prior to isoelectric focusing (IEF) and subsequent SDS-PAGE.

### 2-D DIGE and image analysis

The labeled mixtures were loaded onto Immobiline Dry Strips (24 cm, linear pH gradient from pH 4–7, GE Healthcare). The IPG strips were rehydrated overnight at 40 V for 5 h followed by 100 V for 6 h, and IEF was then conducted for a total of 78 kVhr on a Multiphor II System (GE Healthcare). After completion of the IEF program, the strips were equilibrated and then applied to 12.5% polyacrylamide gels. The SDS-PAGE was performed using Ettan™ Dalt six equipment (GE Healthcare) at 15°C. All electrophoresis procedures were performed in the dark and run in duplicate. Gels were scanned using a Typhoon™ Trio Series Variable Mode Image (GE Healthcare) at 100 µm resolution, followed by silver staining. The resulting gel images were analyzed using DeCyder software 6.5 (GE Healthcare). The biological variation analysis mode (BVA) revealed differences between the PFDoA treated groups and the control across all gels. A Student's *t*-test was used to statistically analyze the data, and *p*<0.05 was considered significant.

### In-gel trypsin digestion and protein identification by MALDI TOF/TOF

Visible differential protein spots were manually excised from the silver-stained gels and placed into a 96-well microtiter plate. Gel pieces were destained with 15 mM potassium ferricyanide and 50 mM sodium thiosulfate (1∶1) for 20 min at room temperature and digested overnight with 12.5 ng/µl trypsin in 20 mM ammonium bicarbonate at 37°C. Peptides were then extracted twice using 0.1% TFA in 50% ACN, dried, and eluted onto the target with 0.7 µl of matrix solution (α-cyano-4-hydroxy-cinnamic acid in 0.1% TFA, 50% ACN). Samples were allowed to air-dry before being analyzed by an ABI 4700 MALDI-TOF/TOF Proteomics Analyzer (Applied Biosystems, Framingham, MA, USA). Positive ion mass spectra were recorded on a home-built linear time-of-flight mass spectrometer using 39 kV of total acceleration energy. Data from the PMF and MALDI-TOF MS/MS were analyzed using MASCOT (Matrix Science, London, UK) search software. The following parameters were used in the search: *Rattus*, protein molecular mass range from 700 to 3,000 Da, trypsin digest with one missed cleavage, peptide tolerance of 0.2, MS/MS tolerance of 0.8 Da, and possible oxidation of methionine. Protein scores (based on combined MS and MS/MS spectra) greater than 56 were considered statistically significant (*p*<0.05). The individual MS/MS spectrum with the statistically significant (confidence interval >95%) best ion score (based on MS/MS spectra) was accepted. The identified proteins were then matched to specific processes or functions by searching Gene Ontology (http://www.geneontology.org/).

### Quantitative PCR and western blot

Five differentially expressed proteins (Table S2) were further examined to detect corresponding mRNA and protein levels in all experimental groups (0, 0.05, 0.2, and 0.5 mg/kg/day PFDoA) by quantitative PCR and western blot, respectively. The detailed methods are given in the Text S1 and Table S2). Hypoxanthine guanine phosphoribosyltransferase 1 (Hprt1) was chosen as an internal control by GeNorm analysis [23]. Differences in mRNA expression levels were calculated using the 2^−ΔΔCt^ method [24]. For western blot analyses and quantitative PCR data, statistical significance was determined using a one-way ANOVA followed by the Duncan multiple range test (SPSS, Inc., Chicago, IL). Data are presented as means with standard errors (mean±SE). A *p*-value of <0.05 was considered statistically significant.

### ^1^H NMR spectroscopy of kidney tissues and data analysis

Kidney samples (∼250 mg) from the control and PFDoA (0.05, 0.2, and 0.5 mg/kg/day) treatments were homogenized in 2 ml of 50% methanol and then centrifuged at 13,000 rpm for 10 min. The supernatant was collected, dried under a stream of nitrogen, and reconstituted in 550 µl of D_2_O. Prior to NMR analysis, 60 µl of 0.1% sodium salt of 3-(trimethylsilyl)propionic-2,2,3,3,-*d4* acid (TSP) in D_2_O was added. Two milliliters of chloroform were added to the pellets (control and 0.5 mg/kg/day PFDoA group), and the extraction was followed by an additional centrifugation. The lipophilic supernatants were removed, dried under a stream of nitrogen, and reconstituted in 600 µl of chloroform-D (containing 0.03% (v/v) tetramethylsilane) prior to NMR analysis. The reconstituted solutions were transferred to 5-mm NMR tubes.

The NMR measurements were performed on a Varian INOVA 600 NMR spectrometer at 599.73 MHz, using a 5-mm triple resonance probe. The 1D NOESY (RD-90°-t1-90°-tm-90°-acquire) pulse sequence was used for water suppression in the tissue extracts. For each sample, 64 transients were collected for a total of 32 K data points. In the lipid extracts, a spectral width of 8,008.0 Hz was acquired with an acquisition time of 2.05 s. In the aqueous extracts, a spectral width of 8,993.8 Hz and an acquisition time of 1.82 s were used. Spectra were manually phased, baseline corrected, and referenced to TMS or TSP at *Δ* 0.0. Total correlation spectroscopy (TOCSY) 2D NMR spectra were also acquired for resonance assignment.

All spectra were manually rephased using the VNMR 6.1C software package (Varian, Inc., Palo Alto, CA). For lipid extracts, the spectra were divided into 135 0.04 ppm-wide segments for a spectral window that ranged from 0.40 to 5.80 ppm. For aqueous extracts, the spectra were reduced to 900 bins of equal width (0.01 ppm) corresponding to the *Δ* 9.40–0.41 region. The segments from *Δ* 5.1–4.7 ppm were removed to eliminate residual water resonance. All remaining spectral segments were scaled to the total integrated area of the spectra to reduce the effects of variation in concentration [25]. The 1D ^1^H NMR spectral data sets for lipid and aqueous extracts were imported into the SIMCA-P10.0 software package (Version 10, Umetrics AB, Umea, Sweden) separately. Partial least squares discriminant analysis (PLS-DA) with mean centering was then applied for data processing.

### Kidney free amino acid profile analyses

Kidney aqueous extracts (40 µl) were mixed with 10 µl of 10% sulfosalicyclic acid using an Apricot TPS-24 automated liquid handler and then centrifuged to precipitate the proteins. Labeling buffer (40 µl) was added to the supernatant, and the resulting mixture was incubated with iTRAQ® Reagent 115 (5 µl) for 30 min at room temperature. A total of 5 µl of 1.2% hydroxylamine solution was added to each sample. Samples were dried and reconstituted with the iTRAQ® Reagent 114-labeled Standard Mix (32 µl). Amino acids were separated and detected by an API 3200™ LC-ESI MS/MS (Applied Biosystems, Framingham, USA) in positive SRM mode. Chromatography was performed using a C18 (150×4.6 mm) column, with mobile phases of 0.1% formic acid: 0.01% heptafluorobutyric acid (HFBA): H_2_O and 0.1% formic acid: 0.01% HFBA: ACN. Total analysis time was 25 min. The concentrations of 42 amino acids were determined by comparing their ion intensity to their respective internal standards. Principal components analysis-discriminant analysis (PCA-DA) was used for data analysis with MarkerView™ Software and pareto scaling (Applied Biosystems, Framingham, USA). It is a supervised multivariate statistical analysis method which combines PCA with DA to facilitate the classification, similar to PLS-DA.

## Results

### 2-D DIGE analysis for kidney proteins

**Figure 1:**
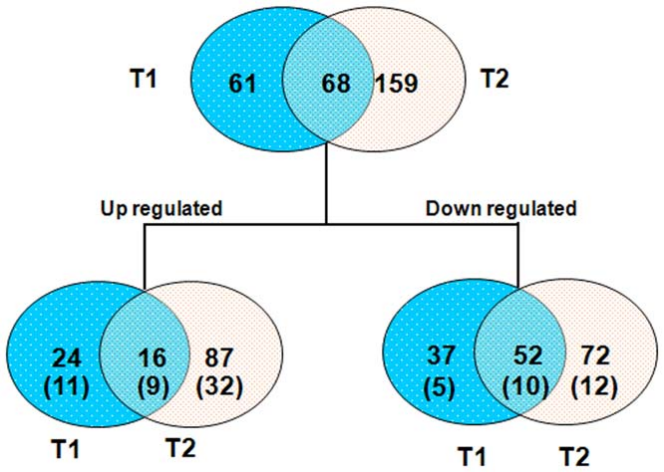
Venn diagram analysis of the differentially expressed proteins in two PFDoA treatment groups. The numbers of differentially expressed spots (up- or down-regulated) in different treatment groups are shown in the different segments (the number of identified proteins is given in brackets). T1, 0.2 mg/kg/day PFDoA group. T2, 0.5 mg/kg/day PFDoA group.

Kidney proteins were separated effectively by 2-D DIGE analysis (Figure S1). Analysis by DeCyder software demonstrated that 129 and 227 spots were significantly altered in the 0.2 and 0.5 mg/kg/day PFDoA groups, respectively, compared to the control rats (*p*<0.05), while 68 proteins (16 up-regulated and 52 down-regulated) were significantly altered in both treatments (Figure 1). One hundred and six protein spots were excised from the silver-stained gels and identified by MALDI-TOF-MS/MS analysis. After a MASCOT database search, 79 different proteins were identified successfully (Figure S1). Some spots were identified as the same protein by TOF/TOF. This overlap might be due to different isoforms or differences in posttranslational modifications.

### Function categories of identified proteins

The 79 identified proteins are summarized and classified according to their biological process or function (Table S3). Twelve of the identified proteins were involved in amino acid metabolism, including 3-mercaptopyruvate sulfurtransferase (3Mpst), 3-phosphoglycerate dehydrogenase (Phgdh), and isovaleryl coenzyme A dehydrogenase (Ivd), which plays an important role in the metabolism of cysteine (Cys), L-serine, valine (Val), leucine (Leu), and isoleucine (Ile). Six proteins were related to the TCA cycle and pyruvate metabolism. As determined by their protein expression levels, malate dehydrogenase 1 (Mdh1), pyruvate carboxylase (PC), pyruvate dehydrogenase (lipoamide) beta (Pdhb), and succinyl-CoA ligase, GDP-forming, subunit beta (Suclg2) were all induced by PFDoA. Eight proteins involved in gluconeogenesis and glycolysis were up-regulated in the kidneys of rats exposed to 0.2 and 0.5 mg/kg/day of PFDoA. Three spots (spot 290, 938, and 965) were identified as fructose-1,6-biphosphatase 1, the key regulatory enzyme of gluconeogenesis. Eight proteins associated with electron transport were all significantly induced at the protein expression level by PFDoA, including NADH dehydrogenase (ubiquinone) Fe-S protein 1 (Ndufs1), ATP synthase (ATP5a1 and ATP5b), and ATPase. Three proteins, Cu-Zn superoxide dismutase (Cu-Zn SOD), peroxiredoxin 3 (Prx3), and thioredoxin reductase (TrxR), involved in the elimination of radical oxygen species (ROS), were up-regulated in the 0.5 mg/kg/day PFDoA treatment. Prohibitin (Phb) and tumor necrosis factor type 1 receptor-associated protein (Trap), which are related to stress response, were also up-regulated.

### Quantitative PCR and western blot validation

**Figure 2:**
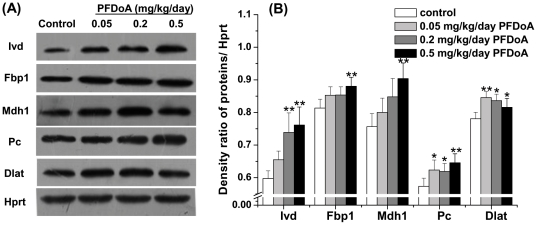
Western blot analysis showing the effect of different PFDoA concentrations on protein expression levels of Ivd, Fbp1, Mdh1, Pc, and Dlat in the kidneys of male rats. Intensities of the proteins were normalized to the corresponding Hprt level. Representative western blots are shown in (A), and the results from densitometry analysis of the western blots are shown in (B). Each bar represents the mean ± SE of six samples per treatment. ^*^ *p*<0.05;^**^ *p*<0.01.

To authenticate the 2-D DIGE proteomic results and verify whether changes in protein expression correlated with transcript level, five proteins related to metabolism (Fbp1, Ivd, Mdh1, Dlat, and Pc) were selected and analyzed by western blot and quantitative PCR. The fold changes in these proteins detected by 2-D DIGE in the 0.2 and 0.5 mg/kg/day PFDoA groups are shown in Table 1. The western blot results followed a similar pattern to the DIGE results; except that the protein expression of Fbp1 in the 0.2 mg/kg/day PFDoA group did not change significantly (*p*>0.05, Figure 2). Similar regulation also occurred at the mRNA expression level. However, the mRNA level of Dlat decreased significantly in the 0.05 mg/kg/day dosage group (*p*<0.05) and did not change in the other two treatments (Figure S2). The low mRNA-protein correlations were also found by other researchers [26], [27]. One of the reasons is the existence of posttranscriptional mechanisms which controls the protein translation rate [28] and the half-lives of specific proteins or mRNAs [29]. These results suggest that PFDoA can have diverse transcriptional regulation effects, but the protein concentration changes of some enzymes were probably related to the change in their transcript levels by PFDoA.

**Table 1:** Protein expression alteration of five selective proteins from 2-D DIGE analysis.

Protein name	Fold change^a^ (treated vs controls)	
	0.2 mg/kg/day	0.5 mg/kg/day
fructose-1,6- biphosphatase 1 (Fbp1)	1.12*	1.01
isovaleryl coenzyme A dehydrogenase (Ivd)	1.06	1.09**
malate dehydrogenase 1 (Mdh1)	1.04	1.08*
dihydrolipoamide S-acetyltransferase (Dlat)	1.08	1.14**
pyruvate carboxylase (Pc)	1.31**	1.32

a The average fold changes were determined by Decyder 6.5.

*Significant difference from control, *p*<0.05.

**Significant difference from control, *p*<0.01. Positive ratios indicate increased expression of proteins in the PFDoA groups compared to control rats.

### ^1^H NMR spectroscopy of kidney tissue

**Figure 3:**
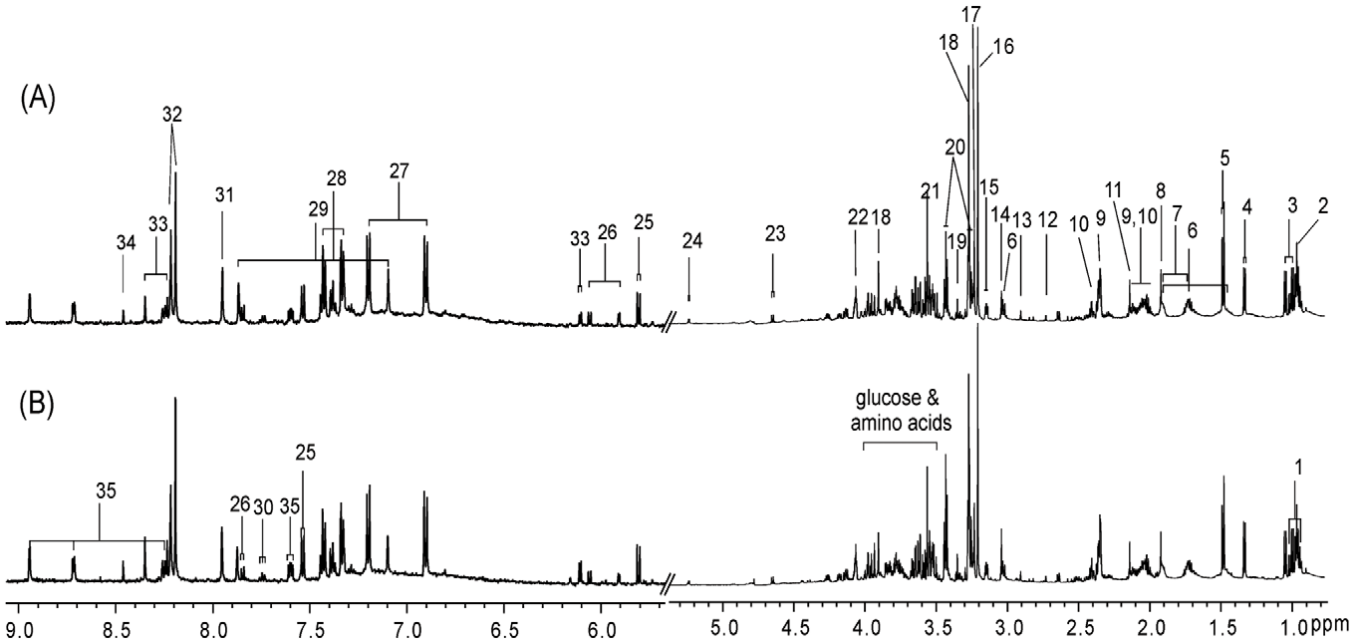
Typical ^1^H NMR spectra of the aliphatic region (0.8–5.4) and aromatic region (δ 5.7–9.1) from aqueous extracts of renal tissues of (A) control rats and (B) rats exposed to 0.5 mg/kg/d PFDoA. The vertical scales of the aromatic regions are enlarged 8 times. Key: 1. isoleucine; 2. leucine; 3. valine; 4. lactate; 5. alanine; 6. lysine; 7. arginine; 8. acetate; 9. glutamate; 10. glutamine; 11. methionine; 12. DMA; 13. DMG; 14. creatine; 15. ethanolamine; 16. choline; 17. phosphorylcholine; 18. betaine; 19. scyllo-inositol; 20. taurine; 21. glycine; 22. myo-inositol; 23. β-glucose; 24. α-glucose; 25. uracil; 26. cytidine; 27. tyrosine; 28. phenylalanine; 29. histidine; 30. tryptophan; 31. xanthine; 32. hypoxanthine; 33. inosine; 34. formate; 35. nicotinamide.

**Figure 4:**
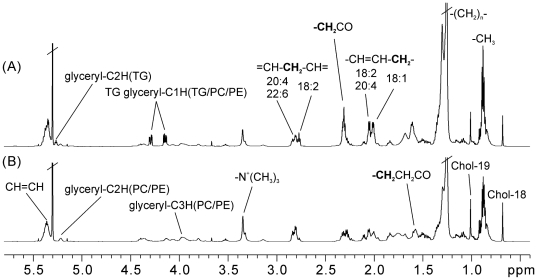
Typical ^1^H NMR spectra of the lipid extracts of renal tissues from (A) control rats and (B) rats exposed to 0.5 mg/kg/d PFDoA. Chol, cholesterol; TG, triglyceride; PC, phosphatidylcholine; PE, phosphatidylethanolamine.

Typical ^1^H NMR spectra of the aqueous and lipid extracts of rats from the control and 0.5 mg/kg dosed group are shown in Figures 3 and 4, respectively. The ^1^H chemical shifts and assignments of the endogenous metabolites were performed according to previous literature and 2D NMR spectra [30]–[33]. The ^1^H NMR spectra of the aqueous tissue extract reflected the lower molecular weight metabolites present in the kidney (Figure 3). These metabolites included amino acids, organic acids, sugars, nucleotides, and their metabolites. The NMR spectra of lipid extracts were dominated by lipid metabolites, which included cholesterol esters, triglycerides, saturated and unsaturated fatty acids, and phosphatides (Figure 4). It was possible to partially assign resonances from mono- and polyunsaturated fatty acids based on references [31].

### ^1^H NMR spectroscopic and pattern recognition analyses of the kidney

**Figure 5:**
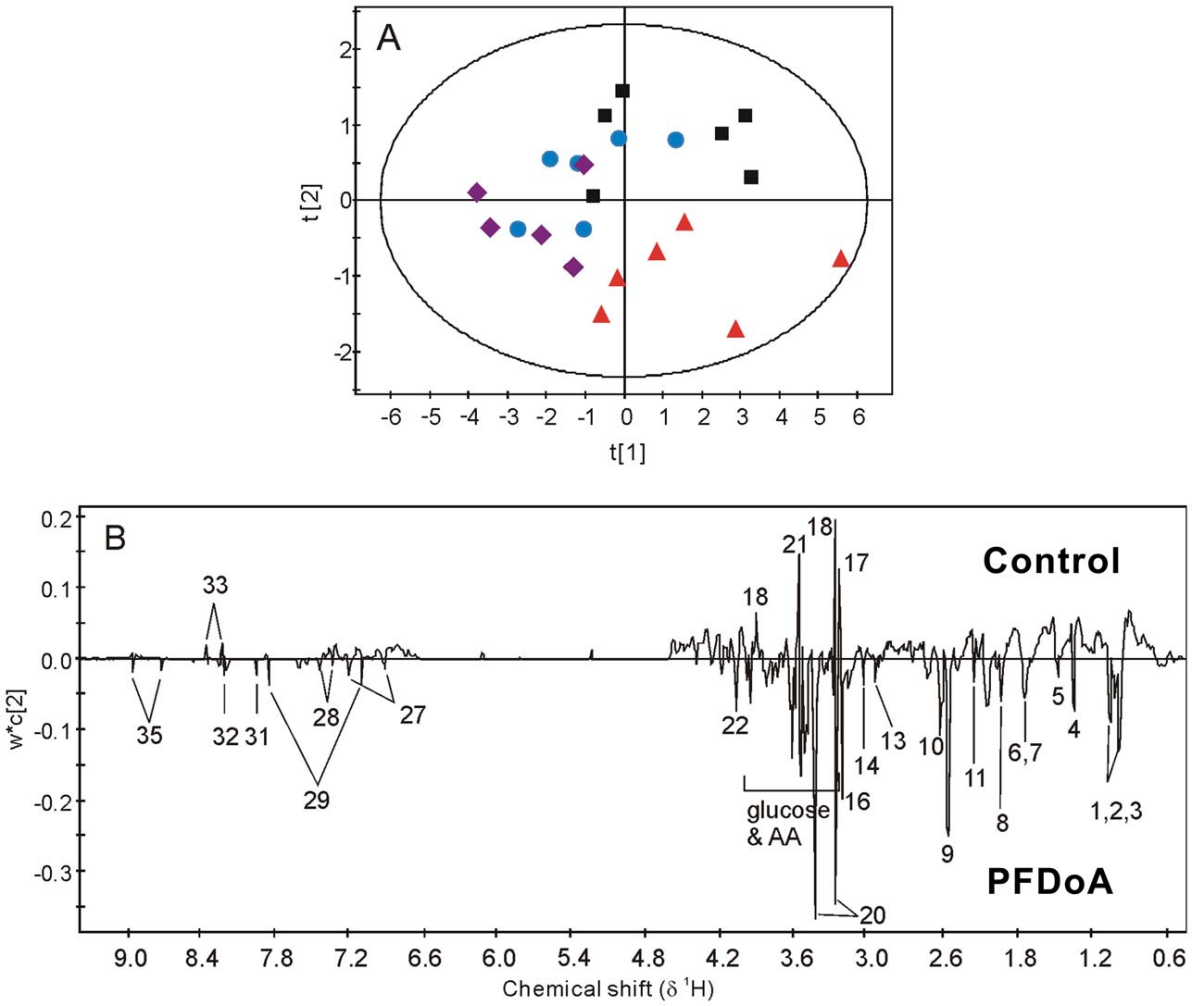
Scores scatter plot and loading line plot of the PLS-DA analysis of aqueous renal tissues extracts from control and PFDoA treated rats. (A) Scores scatter plot for PLS-DA analysis of aqueous renal tissue extracts from control rats (black box ▪) and rats exposed to 0.05 mg/kg/d (blue dot •), 0.2 mg/kg/d (purple diamond ♦) and 0.5 mg/kg/d (red triangle ▴) of PFDoA. (B) The loading line plot illustrates the differentiating metabolites between the treated and control groups. Keys to the metabolites are the same as in Figure 4.

Preprocessing of data, especially normalization of original data is a crucial step in metabonomic studies. Total integral normalization is the most widely used normalization method, and to some extent, is ‘the de facto standard of normalizing NMR spectra’ [34]. This method, however, can fail when there are abrupt changes in the concentrations of some specific groups of metabolites or one specific metabolite, such as in the case of diabetes. There are not strong or abrupt changes of some metabolites in our data sets, thus we used scaled all remaining spectral segments to the total integrated area of the spectra to reduce the effects of variation in concentration [25]. The PLS-DA model was used to compare ^1^H NMR spectra from renal tissue extracts. A dose-dependent tendency for metabolic changes was demonstrated in the scores plot of the NMR data from the aqueous extracts (Figure 5). The line loading plot (Figure 5B) demonstrated the changes in metabolites responsible for the group separation in Figure 5A. Negative peaks represent metabolites with higher concentrations in dosed groups than in the control, whereas positive peaks represent metabolites with higher concentrations in the control than in the dosed groups. Consequently, most metabolite levels were elevated, whereas the concentrations of phosphorylcholine, betaine, glycine, glucose, and inosine decreased in the PFDoA exposed groups (Table 2). There were also some variations in the lipid content of renal tissues in treated rats, as demonstrated in the PLS-DA scores plot of the lipid extract NMR data for the control and 0.5 mg/kg dosed rats (Figure S3). The corresponding loading line plot showed higher content of cholesterol ester, MUFA (except 18∶2), and phosphatidylcholine/phosphatidylethanolamine, and lower content of TG in the 0.5 mg/kg dosed group than in the control group.

**Table 2:** Metabolite changes in renal tissue aqueous extracts from PFDoA treated rats.

Keys	Metabolites	Chemical shift (multiplicity)	Treated vs Control
1	isoleucine	0.95 (t), 1.02 (d)	↑
2	leucine	0.97 (d)	↑
3	valine	0.99 (d), 1.05 (d)	↑
4	lactate	1.33 (d), 4.12 (q)	↑
5	alanine	1.48(d)	↑
6	lysine	1.50(m), 1.73(m), 1.90(m)	↑
7	arginine	1.73(m), 1.90(m)	↑
8	acetate	1.92(s)	↑
9	glutamate	2.35(m),	↑
10	glutamine	2.42(m)	↑
11	methionine	2.14(s)	↑
12	dimethylamine	2.73(s)	-
13	dimethylglycine	2.91(s)	↑
14	creatine	3.04(s)	↑
15	ethanolamine	3.15(t)	-
16	choline	3.21(s)	↑
17	phosphorylcholine	3.23(s)	↓
18	betaine	3.27(s), 3.91(s)	↓
19	scyllo-inositol	3.35(s)	-
20	taurine	3.43(t),3.26(t)	↑
21	glycine	3.56(s)	↓
22	myo-inositol	4.07(m)	↑
23	β-glucose	4.64(d), 3.2-3.9(m)	↓
24	α-glucose	5.23(d), 3.2-3.9(m)	↓
25	uracil	7.54(d),5.81(d)	-
26	cytidine	7.84(d), 6.07(d), 5.92(d)	-
27	tyrosine	7.20(d), 6.90(d)	↑
28	phenylalanine	7.43(t), 7.39(t), 7.33(d)	↑
29	histidine	7.86(s), 7.09(s)	↑
30	tryptophan	7.74(m)	-
31	xanthine	7.95(s)	↑
32	hypoxanthine	8.21(s), 8.19(s)	↑
33	inosine	8.35(s), 8.23(s), 6.10(d)	↓
34	formate	8.46(s)	-
35	nicotinamide	8.94(bs), 8.71(m), 8.25(m)	↑

Note: s, singlet; br, broad single; d, doublet; t, triplet; m, multiplet. The arrows indicate the increase (↑) or decrease (↓) in the levels of metabolites.

### Rat kidney pools of free amino acids in response to different doses of PFDoA

**Figure 6:**
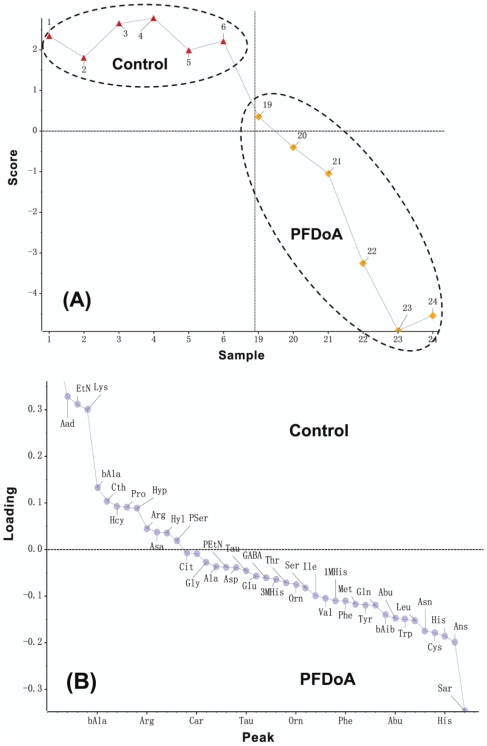
Scores plot (A) and loading plot (B) of the PCA-DA analysis of kidney pools of free amino acids after exposure to 0.5 mg/kg/d PFDoA. The full names of amino acids denoted refer to Table S4.

We used iTRAQ®–LC–MS/MS to measure how kidney amino acids changed with PFDoA exposure. Results demonstrated that PFDoA caused significant alterations in the kidney amino acid profiles based on the analysis of the corresponding loading plot. A marked increase in sarcosine, Asn, His, 1-methylhistidine, Ile, Leu, Val, Trp, Tyr), Phe, Cys, and Met intensity, along with a decrease in the signals of homocitrulline, α-aminoadipic acid, β-alanine, and cystathionine, were responsible for the separation between the 0.5 mg dosed rats and the control group (Figure 6, Table S4).

## Discussion

Perfluorinated carboxylic chemicals, such as PFOA and PFOS, have been shown to induce hepatomegaly and renal hypertrophy in male rats when administered orally at a dose of 5 mg/kg/day for 28 days [35]. In our study, neither the absolute kidney weight nor the relative kidney weight changed after exposure to PFDoA for 110 days (data not shown), which is similar to results obtained by Son *et al*.[18]. There was, however, a clear elevation in serum creatine levels in the 0.2 and 0.5 mg/kg/day PFDoA groups [19], which is a sign of renal insufficiency reflecting lower rates of glomerular filtration [36]. To better understand potential renal toxicity of PFDoA, 2-D DIGE, NMR-based metabonomics, and LC/MS approaches were used in this study. After DIGE and MALDI-MS analyses, 79 differentially expressed proteins were identified between the kidneys of untreated and PFDoA treated rats. These proteins were primarily involved in amino acid metabolism, carbohydrate and energy metabolism, stress response, and electron transport. In addition, the NMR- and LC/MS-based metabolic profile analysis of the kidney samples revealed PFDoA induced perturbation of glucose and amino acid metabolism in the kidney.

At the highest dose of PFDoA exposure, the levels of eight of the twelve differentially expressed proteins related to amino acid metabolism increased significantly. These proteins included Ivd, which converts isovaleryl-CoA to 3-methylcrotonyl-CoA as an intermediate step in the Leu catabolic pathway [37]; Mpst, which plays a central role in cysteine degradation to pyruvate [38]; glutamine transaminase K (Gtk), a freely reversible glutamine (methionine) aromatic amino acid aminotransferase [39]; phenylalanine hydroxylase (Pha), which plays a role in phenylalanine conversion to tyrosine [40]; and L-arginine:glycine amidinotransferase (Agat), which is involved in arginine-related and creatine metabolism [41]. Our NMR and LC/MS-based amino acid profile results showed a marked increase in the levels of branched-chain amino acids (Val, Leu, and Ile), aromatic amino acids (Phe, Trp, and Tyr), and other amino acids, including Asn, His, Gln, Cys, and Met in the 0.5 mg/kg/day PFDoA group, which illustrates kidney dysfunction caused by PFDoA. These increased proteins may contribute to the accelerated amino acid metabolism observed in the kidney after PFDoA treatment. Many of these elevated proteins are also enzymes involved in amino acid biochemistry and hence their metabolic contribution to the observed amino acid profile is an active role. Although catalyzed by different enzymes, the various amino acids are converted to five end products (succinate, oxaloacetate, fumarate, a-ketoglutarate, and pyruvate), which then enter the TCA cycle pathway as substrates.

In this study, increased expression levels of five proteins (Dlat, Pdhb, Pc, Mdh1, and Suclg2) suggested an obvious increase in the rate of the TCA cycle caused by PFDoA. We speculated that this increase was partly caused by accelerated amino acid metabolism, as well as the increase in the level of the amino acid metabolites (lactate, succinate, and pyruvate) in the 0.5 mg/kg/day PFDoA group. Glucose can also be converted into pyruvate through the glycolysis pathway. As Enol and Khk are the two enzymes involved in glycolysis, the up-regulation of these enzymes in protein levels indicates that glycolysis was accelerated in kidneys exposed to PFDoA. In addition, glucogenic amino acids (Val, Leu, Ile, Met, His, Asn, and Cys), lactate, pyruvate, and intermediate metabolites of the TCA cycle can also enter the gluconeogenesis pathway as substrates. Both Pc and Mdh1 play an important role in forming oxaloacetate from pyruvate, and Fbp1 is the key enzyme of gluconeogenesis. The protein levels of the five proteins (Pc, Mdh1, Fbp1, Eno1, and Khk) all increased significantly in the 0.2 and 0.5 mg/kg/day PFDoA groups compared to the control. This indicates that the enrichment of amino acid metabolites and glycolysis accelerated the TCA cycle in the rat kidney. The decrease of glucose in the kidney and increase in intermediates in the TCA cycle and amino acid metabolism also stimulated the gluconeogenesis pathway. In addition, acidosis has been shown to increase renal gluconeogenesis, but impair hepatic gluconeogenesis [42]. Since PFDoA is also a weak acid, it is tempting to speculate that the kidney may be a major factor in accelerating gluconeogenesis upon PFDoA exposure.

In aerobic organisms, various metabolic pathways such as glycolysis, amino acid catabolism, and the TCA cycle are accompanied by energy metabolism. Among the identified proteins, seven were enzymes or subunits related to energy metabolism. Up-regulated proteins such as NADH dehydrogenase, ATP synthase (ATP5a1 and ATP5b), and ATPase, which play important roles in electron transport, showed a significant induction of mitochondrial respiratory function in rat kidneys exposed to PFDoA. Mitochondrial respiratory activity is always accompanied by the production of ROS, which may cause mitochondrial DNA damage or cell death [43], In this study, the protein levels of antioxidant enzymes including Cu-Zn SOD, Prdx3, and TrxR all increased, which suggests an increased production of ROS in the kidney. In addition, the classic “heat shock” chaperones grp75 (Hspa9a) and Hsp60 (Hspd1), which are associated with the mitochondria [44], were up-regulated. This result is similar to that obtained in a previous study by Witzmann *et al*. [45] These molecular chaperones can regulate protein turnover and assembly and protect cells from harmful conditions, including oxidative stress [46]. Coates *et al*. reported that prohibitin could be induced by metabolic stress and may play a role in regulating mitochondrial respiratory activity [47]. Some researchers have also found that the expression of prohibitin increased in apoptotic cells [48], [49]. Therefore, the observed up-regulation of these proteins in this study exhibited a preferential effect of PFDoA on the mitochondria, which might be induced by the increase in the metabolism of amino acids, carbohydrates, and energy in rat kidneys exposed to PFDoA. Additionally, taurine is a ß-amino acid and antioxidant agent naturally found in the kidney that assists with protection [50], [51]. The increase in taurine concentration observed during metabolic analysis may be a complementary reaction to PFDoA-induced oxidative damage in the kidney. Moreover, elevated Myo-inositol, a renal medullary osmolyte, is a marker of renal medullary injury [52].

Perfluorinated carboxylic chemicals are mainly accumulated in the liver and kidneys of animals. Han *et al*. found that 70% of PFOA are distributed in the cytosolic fraction of the rat kidney, and 40% of PFOA in male kidney cytosol are protein bound [53]. Organic anion transporters and some binding proteins, including fatty acid binding protein and α_2u_ globulin (A2Us), were presumed to play an important role in the elimination and reabsorption of PFOA in rat kidneys [54], [55]. In addition, A2Us are well known male rat-specific proteins and include the liver-form and kidney-form α_2u_-globulins (A2U_L_ and A2U_K_). They are capable of binding PFOA *in vitro*, although the binding affinity is relatively weak [56]. The globulin A2U_L_ is synthesized exclusively in the liver of adult male rats [57], secreted into the blood stream, and freely filtered by the glomerulus [58]. Approximately 50% of the filtered proteins are excreted in urine and form the major component of male rat urinary proteins. The balance is reabsorbed by the epithelial cells of the proximal tubule [59], [60]. Reabsorbed A2U_L_ can be degraded into amino acids and undergo limited proteolysis to form A2U_K_. In the present study, four protein spots were identified as α_2u_ globulin PGCL1 (A2U_L1_, two spots), α_2u_ globulin PGCL2 (A2U_L2_), and major urinary protein 5 (MUP5). Levels of these proteins decreased significantly in both 0.2 and 0.5 mg/kg/day PFDoA groups, the maximum being 5.95-fold that of the control. Ciprofibrate, which is a peroxisome proliferator, represses the amount of α_2u_-globulin mRNA and protein in the male rat liver [61]. In our previous study, PFDoA was also identified as a peroxisome proliferator [19], [62]. The decrease of A2Us in the kidney was possibly due to the repression of A2U synthesis in the liver in response to PFDoA. The significant decrease in A2Us could prevent the reabsorption of PFDoA in the proximal tubule, and consequently slow accumulation of PFDoA in rat kidneys.

Several limitations of “-omic” techniques as used in the present study need to be noted. First, some low-abundance proteins, basic proteins or insoluble membrane-associated proteins may not be detected using 2-D DIGE analysis. Then, we examined protein expression from the whole kidney, which could not identify the localized changes such as those that may be confined to glomeruli or tubules. Although this can give some information about renal dysfunction from perturbed proteins and metabolites and reduce the system errors as much as possible; however, this may affect the magnitude of changes in individual intrarenal structures. In addition, we examined renal protein expression at only one time-point, which did not represent the entire dynamic process of PFCs nephrotoxicity. Finally, due to the complexity of biological processes in organisms, including feedforward, feedback and open loop, the detail causality of the changes could not be clarified from one or several “-omics” research *in vivo*. But what we could do was to put forward a potential toxicity and the probable consequences of effects as far as possible in this study, as well as yield new insights into PFCs nephrotoxictity. Therefore, further studies including *in vitro* and *in vivo* tests, detection of time-courses of effects and changes in individual regions of kidneys would shed more light on the definite proteins' functional roles and the consequence of these effects observed herein.

In summary, this paper reports the renal toxicity induced by PFDoA as determined by proteomics and NMR- and LC/MS-based metabonomics. The changes in the proteomic and metabonomic profiles showed that PFDoA could cause kidney damage, primarily perturbing kidney glucose and amino acid metabolism and inducing mitochondrial disorders and oxidative stress to the kidney. Furthermore, the decrease in A2Us possibly prevents PFDoA-induced nephrotoxicity by repressing reabsorption of PFDoA in the proximal tubule and decelerating PFDoA accumulation in the kidney. Our study provides information on the broad and potential effect of PFDoA on the kidney based on levels of proteins and metabolites.

## Supporting Information

Figure S1 **2-D DIGE gray-scale image of kidney protein expression (Cy2-labeled internal standard).** The 79 successfully identified, differentially expressed proteins are indicated with boxes containing the master number.
(DOCX)

Figure S2 **Quantitative PCR analysis of renal mRNA expression levels of Ivd, Fbp1, Mdh1, Pc, and Dlat from control and PFDoA-exposed male rats.** Gene expression levels represent the relative mRNA expression compared to the Hprt levels. Values indicate the mean±SE for six rats per group. ^*^ *p*<0.05; ^**^ *p*<0.01.
(DOCX)

Figure S3 **Scores plot (A) and loading line plot (B) of the PLS-DA analysis of lipid renal tissue extracts from control rat (black box) and rats exposed to 0.5 mg/kg/d (blue dot) of PFDoA.**
(DOCX)

Table S1 **Experimental design for DIGE analysis.** The internal Standard (IS) was the pooled sample using an equal amount of nine experimental samples; A1–A3 were protein samples of control, B1–B3 were protein samples of 0.2 mg/kg/day PFDoA groups, C1–C3 were protein samples of 0.5 mg/kg/day PFDoA groups. Each experimental sample was pooled from two independent protein extracts in the same group.
(DOCX)

Table S2 **Sequences of primers used for quantitative PCR amplification.**
(DOCX)

Table S3 **Classification of the differentially expressed proteins identified in the kidney of rats exposed to PFDoA compared with the control.**
(DOCX)

Table S4 **Fold changes of free amino acids in renal tissue detected by iTRAQ®–LC –MS/MS.**
(DOCX)

Text S1 **Protocols of quantitative PCR and western blot.**
(DOC)
