# Supplementary material for: Biological Responses to Perfluorododecanoic Acid Exposure in Rat Kidneys as Determined by Integrated Proteomic and Metabonomic Studies
Source: PLoS One. 2011 Jun 3;6(6):e20862. doi: 10.1371/journal.pone.0020862 (PMC3108999; doi:10.1371/journal.pone.0020862)
Supplement: Figure S1 — 2-D DIGE gray-scale image of kidney protein expression (Cy2-labeled internal standard). The 79 successfully identified, differentially expressed proteins are indicated with boxes containing the master number. (DOCX) [file pone.0020862.s001.docx]

**Figure S1.** 2-D DIGE gray-scale image of kidney protein expression (Cy2-labeled internal standard). The 79 successfully identified, differentially expressed proteins are indicated with boxes containing the master number.

******
