# Supplementary material for: Biological Responses to Perfluorododecanoic Acid Exposure in Rat Kidneys as Determined by Integrated Proteomic and Metabonomic Studies
Source: PLoS One. 2011 Jun 3;6(6):e20862. doi: 10.1371/journal.pone.0020862 (PMC3108999; doi:10.1371/journal.pone.0020862)
Supplement: Figure S2 — Quantitative PCR analysis of renal mRNA expression levels of Ivd, Fbp1, Mdh1, Pc, and Dlat from control and PFDoA-exposed male rats. Gene expression levels represent the relative mRNA expression compared to the Hprt levels. Values indicate the mean±SE for six rats per group. * p<0.05; ** p<0.01. (DOCX) [file pone.0020862.s002.docx]

**Figure S2.** Quantitative PCR analysis of renal mRNA expression levels of Ivd, Fbp1, Mdh1, Pc, and Dlat from control and PFDoA-exposed male rats. Gene expression levels represent the relative mRNA expression compared to the Hprt levels. Values indicate the mean ± SE for six rats per group. ^*^*p* < 0.05; ^**^*p* < 0.01.

******
