# Supplementary material for: Biological Responses to Perfluorododecanoic Acid Exposure in Rat Kidneys as Determined by Integrated Proteomic and Metabonomic Studies
Source: PLoS One. 2011 Jun 3;6(6):e20862. doi: 10.1371/journal.pone.0020862 (PMC3108999; doi:10.1371/journal.pone.0020862)

**Figure S3.**Scores plot (A) and loading line plot (B) of the PLS-DA analysis of renal tissue lipid extracts from control rat (black box) and rats exposed to 0.5 mg/kg/d (blue dot) of PFDoA.


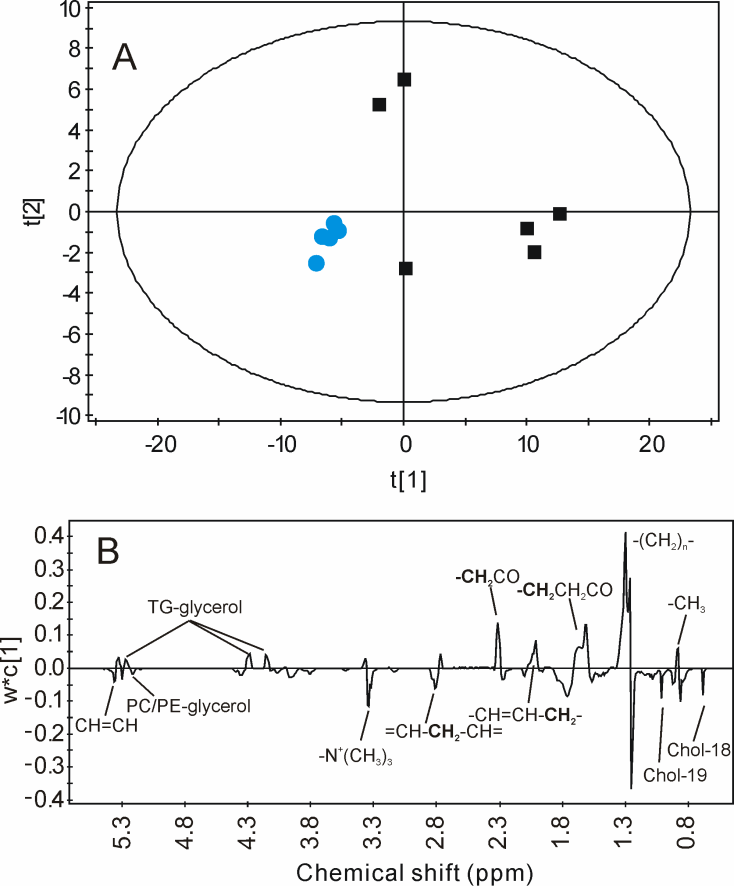

Supplement: Figure S3 — Scores plot (A) and loading line plot (B) of the PLS-DA analysis of lipid renal tissue extracts from control rat (black box) and rats exposed to 0.5 mg/kg/d (blue dot) of PFDoA. (DOCX) [file pone.0020862.s003.docx]
