# Supplementary material for: Biological Responses to Perfluorododecanoic Acid Exposure in Rat Kidneys as Determined by Integrated Proteomic and Metabonomic Studies
Source: PLoS One. 2011 Jun 3;6(6):e20862. doi: 10.1371/journal.pone.0020862 (PMC3108999; doi:10.1371/journal.pone.0020862)
Supplement: Table S1 — Experimental design for DIGE analysis. The internal Standard (IS) was the pooled sample using an equal amount of nine experimental samples; A1–A3 were protein samples of control, B1–B3 were protein samples of 0.2 mg/kg/day PFDoA groups, C1–C3 were protein samples of 0.5 mg/kg/day PFDoA groups. Each experimental sample was pooled from two independent protein extracts in the same group. (DOCX) [file pone.0020862.s004.docx]

**Table S1.** Experimental design for 2-D-DIGE analysis

|  |  | | |
| --- | --- | --- | --- |
| **Gel no.** | **CyDye^TM^ DIGE Fluor minimal dyes** | | |
|  | **Cy2** | **Cy3** | **Cy5** |
| 1 | IS^a^ | A1 ^b^ | B1 |
| 2 | IS | B2 | C1 |
| 3 | IS | C2 | A2 |
| 4 | IS | A3 | C3 |
| 5 | IS | B3 | A1 |

^a^ Internal Standard (IS) pooled from equal amount of nine experimental samples; ^b^ A1-A3 were protein samples of control, B1-B3 were protein samples of 0.2 mg/kg/day PFDoA groups, C1-C3 were protein samples of 0.5 mg/kg/day PFDoA groups. Each experimental sample was pooled from two independent protein extracts in the same group. Gels were loaded with paired samples according to the Table S1. .
