# Supplementary material for: Biological Responses to Perfluorododecanoic Acid Exposure in Rat Kidneys as Determined by Integrated Proteomic and Metabonomic Studies
Source: PLoS One. 2011 Jun 3;6(6):e20862. doi: 10.1371/journal.pone.0020862 (PMC3108999; doi:10.1371/journal.pone.0020862)
Supplement: Table S2 — Sequences of primers used for quantitative PCR amplification. (DOCX) [file pone.0020862.s005.docx]

**Table S2**. Sequences of primers used for quantitative PCR amplification

|  |  |  |  |
| --- | --- | --- | --- |
| **Gene** | **GenBank Accession No.^a^** | **5’→3’ Primer Sequences ^b^** | **Product Length (bp)** |
| Hprt1 | NM_012583 | F: GTTGGATACAGGCCAGAC  R: TCCACTTTCGCTGATGAC | 123 |
| Actb | NM_031144 | F: TCGTGCGTGACATTAAAGAG  R: ATTGCCGATAGTGATGACCT | 134 |
| Gapd | NM_017008 | F: TGGAGTCTACTGGCGTCTT  R: GCTGACAATCTTGAGGGAG | 158 |
| Ywhaz | BC094305 | F: AGGGACATCTGCAACGAC  R: CAGCAACCTCAGCCAAGT | 130 |
| Hmbs | NM_013168 | F: GATGTGCCTACCATACTACCT  R: CCAGGGTCTTTCCAATAA | 109 |
| Pc | NM_012744 | F: GCATCCGCCTGGACAAT  R: TGCCCGCTAGGAACTGC | 213 |
| Dlat | NM_031025 | F: CTCCTATCCCGTTCACAT  R: TCTGCCAGCAAGTCTCC | 123 |
| Fbp1 | NM_012558 | F: TTTTGGTGGACAGGGATG  R: CCAGAGTGCGGTGAACAT | 189 |
| Ivd | NM_012592 | F: CCTGCTGCTAACATCCTG  R: CTTCCCTCACATGCAAATA | 151 |
| Mdh1 | BC059124 | F: CATCAAGGCTCGGAAGC  R: CATCAGGGACACCATAGGA | 146 |

^a^ Genbank accession number (<http://www.nvbi.nlm.nih.gov>) used to design the primers. ^b^ F: forward primer; R: reverse primer. Hprt: hypoxanthine guanine phosphoribosyl transferase 1; Actb: β-actin; Gapd: glyceraldehyde-3-phosphate dehydrogenase; Ywhaz: tyrosine 3-monooxygenase/ tryptophan, 5-monooxygenase activation protein, zeta; Hmbs: hydroxymethylbilane synthase; Dlat: dihydrolipoamide S-acetyltransferase; Fbp1: fructose-1,6- biphosphatase 1; Ivd: isovaleryl coenzyme A dehydrogenase; Mdh1: malate dehydrogenase 1.
