# Supplementary material for: Biological Responses to Perfluorododecanoic Acid Exposure in Rat Kidneys as Determined by Integrated Proteomic and Metabonomic Studies
Source: PLoS One. 2011 Jun 3;6(6):e20862. doi: 10.1371/journal.pone.0020862 (PMC3108999; doi:10.1371/journal.pone.0020862)
Supplement: Table S3 — Classification of the differentially expressed proteins identified in the kidney of rats exposed to PFDoA compared with the control. (DOCX) [file pone.0020862.s006.docx]

|  |  |  |  |  |  |  | | | |
| --- | --- | --- | --- | --- | --- | --- | --- | --- | --- |
| **Spot No.^a^** | **Protein name** | **GI no.^b^** | **Theor.**  **MW/PI** | **%**  **SC.^e^** | **Protein score** | **Fold change and T-test value^c^** | | | |
|  |  |  |  |  |  | **0.2 mg/kg/day** | | **0.5 mg/kg/day** | |
|  |  |  |  |  |  | **Ratio** | **p value** | **Ratio** | **p value** |
| **Metabolism^d^ (29)** | |  |  |  |  |  |  |  |  |
| ***Amino acid metabolism* (12)** | |  |  |  |  |  |  |  |  |
| 1237 | 3-mercaptopyruvate sulfurtransferase | gi\|20304123 | 32919.4/5.88 | 25 | 147 | 1.07 | 0.23 | 1.11 | 0.048 |
| 551 | 3-phosphoglycerate dehydrogenase | gi\|13928850 | 56457.2/6.28 | 3 | 60 | 1.07 | 0.28 | 1.14 | 0.011 |
| 1542 | Chain, Catechol O-methyltransferase | gi\|1633081 | 24731.4/5.11 | 23 | 155 | 1.11 | 0.68 | 1.1 | 0.0029 |
| 841 | Creatine kinase | gi\|203474 | 42699.3/5.33 | 25 | 132 | -1.08 | 0.26 | -1.15 | 0.038 |
| 1112 | Crystallin, mu | gi\|16758840 | 33533.1/5.34 | 18 | 136 | 1.03 | 0.55 | 1.18 | 0.0059 |
| 897 | Isovaleryl coenzyme A dehydrogenase | gi\|6981112 | 46405.8/8.03 | 49 | 171 | 1.06 | 0.082 | 1.09 | 0.0033 |
| 814 | Kynurenine aminotransferase; glutamine transaminase K; | gi\|68825 | 47742.8/6.22 | 18 | 83 | 1.16 | 0.00072 | 1.08 | 0.023 |
| 751 | L-arginine: glycine amidinotransferase | gi\|13591949 | 48211/7.17 | 28 | 213 | 1.04 | 0.29 | 1.15 | 0.00055 |
| 717 | Phenylalanine hydroxylase | gi\|50926235 | 51787.3/5.76 | 27 | 238 | 1.18 | 0.029 | 1.22 | 0.0026 |
| 658 | Plasma glutamate carboxypeptidase | gi\|47477878 | 52008.6/5.99 | 31 | 195 | -1.11 | 0.037 | -1.12 | 0.0052 |
| 1059 | Dimethylarginine dimethylaminohydrolase 1 | gi\|11560131 | 31405.9/5.75 | 38 | 202 | 1.05 | 0.24 | 1.14 | 0.0038 |
| 1460 | PREDICTED: similar to indolethylamine N-methyltransferase | gi\|62647320 | 29475.8/5.74 | 15 | 69 | -1.67 | 0.00055 | -1.39 | 0.0097 |
| ***TCA cycle and pyruvate metabolism* (6)** | | |  |  |  |  |  |  |  |
| 1161 | Malate dehydrogenase 1, NAD (soluble) | gi\|37590235 | 36461/5.93 | 36 | 263 | 1.04 | 0.27 | 1.08 | 0.035 |
| 896 | PREDICTED: similar to succinate-coenzyme A ligase, ADP-forming, beta subunit | gi\|62661722 | 50274.1/7.57 | 28 | 195 | 1.1 | 0.015 | 1.2 | 2.50E-05 |
| 344 | Propionyl-CoA carboxylase alpha chain precursor - rat | gi\|92654 | 77562.9/6.33 | 38 | 379 | 1.09 | 0.00069 | 1.17 | 0.031 |
| 1147 | Pyruvate dehydrogenase (lipoamide) beta | gi\|56090293 | 38957/6.2 | 31 | 217 | 1.04 | 0.053 | 1.11 | 0.00012 |
| 418 | Dihydrolipoamide S-acetyltransferase | gi\|78365255 | 67123.4/8.76 | 11 | 168 | 1.08 | 0.058 | 1.14 | 7.50E-05 |

**Table S3.** Classification of the differentially expressed proteins identified in the kidney of rats exposed to PFDoA compared with the control

| (Continued) | | | | | | | | | |
| --- | --- | --- | --- | --- | --- | --- | --- | --- | --- |
| 926 | Succinyl-CoA ligase, GDP-forming, subunit beta | gi\|59808474 | 41694.8/5.64 | 33 | 266 | 1.05 | 0.15 | 1.12 | 0.011 |
| ***Gluconeogenesis and glycolysis* (8)** | |  |  |  |  |  |  |  |  |
| 632 | 4-trimethylaminobutyraldehyde dehydrogenase (TMABADH) (aldehyde dehydrogenase 9A1) | gi\|78099320 | 53618/6.57 | 21 | 162 | 1.14 | 0.51 | 1.63 | 0.04 |
| 84 | Pyruvate carboxylase | gi\|31543464 | 129694.7/6.34 | 25 | 352 | 1.31 | 0.0066 | 1.32 | 0.071 |
| 729 | Enolase 1, alpha | gi\|59808815 | 47128.3/6.16 | 33 | 241 | 1.03 | 0.74 | 1.11 | 0.0091 |
| 757 | Enolase 1, alpha | gi\|59808815 | 47128.3/6.16 | 36 | 152 | 1.08 | 0.33 | 1.1 | 0.033 |
| 290 | Fructose-1,6- biphosphatase 1 | gi\|51036635 | 39584.1/5.54 | 44 | 354 | 1.12 | 0.037 | 1.01 | 0.92 |
| 938 | Fructose-1,6- biphosphatase 1 | gi\|51036635 | 39584.1/5.54 | 38 | 364 | 1.1 | 0.0049 | 1.02 | 0.43 |
| 965 | Fructose-1,6- biphosphatase 1 | gi\|51036635 | 39584.1/5.54 | 55 | 377 | 1.14 | 0.0043 | 1.06 | 0.24 |
| 1184 | Ketohexokinase | gi\|13994119 | 32728.5/6.24 | 20 | 101 | 1.06 | 0.028 | 1.05 | 0.17 |
| ***Lipid metabolism* (1)** | |  |  |  |  |  |  |  |  |
| 1156 | Crystallin, lambda 1 | gi\|28461157 | 35318/5.94 | 10 | 307 | 1.11 | 0.048 | 1.17 | 0.0048 |
| ***Other metabolism* (2)** | |  |  |  |  |  |  |  |  |
| 848 | Ectonucleoside triphosphate diphosphohydrolase 5 | gi\|40786479 | 47342.8/5.08 | 15 | 145 | -1.01 | 0.78 | -1.12 | 0.036 |
| 1107 | Pyridoxal (pyridoxine, vitamin B6) kinase | gi\|13929082 | 34885.6/6.32 | 27 | 212 | 1.07 | 0.16 | 1.16 | 0.0037 |
| **Signal transduction (6)** | |  |  |  |  |  |  |  |  |
| 455 | Alpha isoform of regulatory subunit A, protein phosphatase 2 | gi\|8394027 | 65280.9/5 | 10 | 103 | -1.11 | 0.0035 | -1.07 | 0.051 |
| 613 | ERM-binding phosphoprotein | gi\|11024674 | 38806.6/5.7 | 27 | 147 | -1.3 | 0.084 | -1.44 | 0.026 |
| 620 | ERM-binding phosphoprotein | gi\|11024674 | 38806.6/5.7 | 25 | 93 | -1.28 | 0.0074 | -1.58 | 5.90E-05 |
| 770 | Heterogeneous nuclear ribonucleoprotein F | gi\|19527048 | 45700.9/5.31 | 34 | 60 | 1.03 | 0.55 | 1.09 | 0.014 |
| 437 | Heterogeneous nuclear ribonucleoprotein K | gi\|13384620 | 50944.4/5.38 | 9 | 111 | -1.07 | 0.17 | -1.17 | 0.017 |
| 1397 | Rho GDP dissociation inhibitor (GDI) alpha | gi\|31982030 | 23392.8/5.12 | 33 | 146 | -1.08 | 0.11 | -1.08 | 0.017 |
| **Electron transport (8)** | |  |  |  |  |  |  |  |  |
| 261 | NADH dehydrogenase (ubiquinone) Fe-S protein 1, 75kDa | gi\|53850628 | 79361.6/5.65 | 25 | 241 | 1.04 | 0.56 | 1.19 | 0.0073 |
| 1456 | PREDICTED: similar to NADH dehydrogenase (ubiquinone) Fe-S protein 3 | gi\|27702072 | 30207.6/7.07 | 28 | 268 | 1.11 | 0.055 | 1.07 | 0.0034 |
| 1537 | PREDICTED: similar to NADH dehydrogenase (ubiquinone) Fe-S protein 8 | gi\|27661165 | 23954.7/5.87 | 56 | 118 | 1.06 | 0.033 | 1.07 | 0.025 |

| (Continued) | | | | | | | | | |
| --- | --- | --- | --- | --- | --- | --- | --- | --- | --- |
| 616 | ATP synthase, H^+^ transporting, mitochondrial F1 complex, alpha subunit, isoform 1 | gi\|40538742 | 59716.6/9.22 | 29 | 178 | 1.11 | 0.14 | 1.18 | 0.00048 |
| 680 | ATP synthase, H^+^ transporting, mitochondrial F1 complex, beta subunit | gi\|54792127 | 56318.5/5.19 | 40 | 581 | 1.03 | 0.6 | 1.13 | 0.00017 |
| 1073 | ATPase, H^+^ transporting, V0 subunit D isoform 1 | gi\|31981304 | 40275.2/4.89 | 9 | 76 | 1.1 | 0.015 | 1.04 | 0.35 |
| 1303 | Prohibitin | gi\|6679299 | 29801.9/5.07 | 47 | 384 | 1.02 | 0.84 | 1.14 | 0.026 |
| 625 | Vacuolar H^+^ ATPase B2 | gi\|17105370 | 56514.9/5.57 | 31 | 192 | 1.09 | 0.014 | 1.02 | 0.75 |
| **Stress response (10)** | |  |  |  |  |  |  |  |  |
| 1675 | Cu-Zn superoxide dismutase | gi\|203658 | 15699.7/5.88 | 39 | 273 | 1.07 | 0.4 | 1.15 | 0.027 |
| 522 | Heat shock protein 1 | gi\|11560024 | 60927.4/5.91 | 29 | 338 | 1.12 | 0.064 | 1.26 | 1.80E-05 |
| 236 | Inner membrane protein, mitochondrial | gi\|77917546 | 67135.2/5.57 | 62 | 201 | 1.05 | 0.28 | 1.1 | 0.045 |
| 1508 | Peroxiredoxin 3 | gi\|11968132 | 28303.5/7.14 | 16 | 147 | 1 | 0.93 | 1.09 | 0.02 |
| 320 | PREDICTED: similar to stress-70 protein, mitochondrial precursor (75 kDa glucose regulated protein) (GRP 75) | gi\|62664205 | 73698.8/5.87 | 30 | 518 | 1.1 | 0.039 | 1.22 | 0.00083 |
| 523 | Protein disulfide-isomerase A3 precursor (Disulfide isomerase ER-60) (ERp60) | gi\|1352384 | 56587.7/5.88 | 39 | 386 | -1.64 | 1.40E-05 | -1.13 | 0.27 |
| 530 | Protein disulfide-isomerase A3 precursor (Disulfide isomerase ER-60) (ERp60) | gi\|1352384 | 56587.7/5.88 | 29 | 269 | 1.75 | 4.90E-05 | 1.39 | 0.12 |
| 536 | Protein disulfide-isomerase A3 precursor (Disulfide isomerase ER-60) (ERp60) | gi\|1352384 | 56587.7/5.88 | 17 | 140 | 1.55 | 0.0052 | 1.24 | 0.18 |
| 537 | Thioredoxin reductase | gi\|78191795 | 54622.7/6.07 | 18 | 142 | 1.06 | 0.13 | 1.12 | 0.01 |
| 355 | Tumor necrosis factor type 1 receptor-associated protein | gi\|84781723 | 80410.8/6.56 | 19 | 153 | 1.09 | 0.058 | 1.23 | 0.0058 |
| **Cytoskeleton (7)** | |  |  |  |  |  |  |  |  |
| 873 | Actin beta - rat | gi\|71620 | 41723.7/5.29 | 30 | 273 | -1.22 | 0.024 | -1.21 | 0.021 |
| 881 | Actin beta - rat | gi\|71620 | 41723.7/5.29 | 47 | 382 | -1.07 | 0.026 | -1.03 | 0.098 |
| 884 | Actin beta - rat | gi\|71620 | 41723.7/5.29 | 47 | 376 | -1.21 | 0.065 | -1.21 | 0.037 |
| 341 | Moesin | gi\|1354068 | 67696.8/6.16 | 20 | 116 | 1.1 | 0.27 | 1.32 | 0.0063 |
| 345 | Thrombin | gi\|207304 | 27385/9.38 | 35 | 67 | 1.05 | 0.23 | 1.06 | 0.05 |
| 1300 | Tropomyosin 4 | gi\|6981672 | 28492.5/4.66 | 25 | 156 | -1.12 | 0.086 | -1.18 | 0.005 |
| 528 | Tubulin, alpha 6 | gi\|58865558 | 49905.5/4.69 | 49 | 223 | -1.05 | 0.48 | -1.15 | 0.027 |

| (Continued) | | | | | | | | | |
| --- | --- | --- | --- | --- | --- | --- | --- | --- | --- |
| **Transport (8)** | |  |  |  |  |  |  |  |  |
| 1659 | Alpha-2u globulin PGCL1 | gi\|22219450 | 20723.5/5.85 | 53 | 299 | -1.79 | 0.009 | -3.77 | 1.80E-05 |
| 1668 | Alpha-2u globulin PGCL1 | gi\|22219450 | 20723.5/5.85 | 17 | 210 | -2.31 | 0.00037 | -5.95 | 1.50E-05 |
| 1655 | Alpha-2u globulin PGCL2 | gi\|54020747 | 20711.4/5.62 | 16 | 111 | -1.52 | 0.071 | -4.24 | 0.00022 |
| 1670 | Major urinary protein 5 | gi\|42627893 | 20798.6/5.97 | 14 | 130 | -1.88 | 0.0016 | -4.74 | 2.50E-05 |
| 346 | Hemopexin | gi\|60688311 | 51318.3/7.58 | 10 | 120 | 1.2 | 0.039 | 1.35 | 0.068 |
| 1351 | Intracellular chloride ion channel protein p64H1 | gi\|13929166 | 28614.7/5.92 | 17 | 60 | 1.1 | 0.029 | 1.02 | 0.56 |
| 596 | Selenium-binding protein 2 | gi\|18266692 | 52498.4/6.1 | 27 | 255 | 1.02 | 0.53 | 1.09 | 0.015 |
| 612 | Selenium-binding protein 2 | gi\|18266692 | 52498.4/6.1 | 32 | 244 | 1.04 | 0.23 | 1.09 | 0.013 |
| **Other functions (12)** | |  |  |  |  |  |  |  |  |
| 1546 | Abhydrolase domain containing 14b | gi\|56090461 | 22603.7/5.65 | 30 | 130 | -1.07 | 0.008 | 1.01 | 0.91 |
| 1264 | Chain, Annexin V | gi\|1421099 | 35401.2/4.97 | 48 | 330 | -1.24 | 0.019 | -1.23 | 0.0052 |
| 430 | Dihydropyrimidinase-like 2 | gi\|40254595 | 62238.6/5.95 | 8 | 100 | -1.08 | 0.25 | -1.29 | 0.0004 |
| 558 | Iodothyronine 5' monodeiodinase | gi\|202549 | 54033.2/4.87 | 19 | 156 | -1.12 | 0.017 | -1.02 | 0.44 |
| 561 | Iodothyronine 5' monodeiodinase | gi\|202549 | 54033.2/4.87 | 16 | 114 | -1.21 | 0.0053 | -1.09 | 0.01 |
| 1327 | MAWD-binding protein | gi\|51491893 | 31667.1/5.9 | 17 | 169 | 1.06 | 0.29 | 1.15 | 0.013 |
| 842 | N-myc downstream regulated gene 1 | gi\|58865550 | 42926.9/5.76 | 31 | 175 | 1.16 | 0.049 | 1.26 | 0.048 |
| 903 | PREDICTED: hypothetical protein | gi\|62650801 | 37890.2/6.05 | 28 | 112 | 1.07 | 0.22 | 1.12 | 0.036 |
| 460 | PREDICTED: similar to Protein C14orf159, mitochondrial precursor | gi\|62651145 | 66014/6.21 | 12 | 122 | 1.05 | 0.32 | -1.07 | 0.049 |
| 1518 | Proteasome (prosome, macropain) subunit, beta type 4 | gi\|13928866 | 25777.9/6.33 | 22 | 73 | -1.08 | 0.38 | -1.19 | 0.015 |
| 191 | Valosin-containing protein | gi\|17865351 | 89292.7/5.14 | 23 | 124 | -1.03 | 0.66 | -1.13 | 0.037 |

^a^ Unique spot number of the position where the spot is displayed in the master gel.

^b^ Accession number according to the NCBI rat database.

^c^ Fold change and *p*-value (*t*-test) between the PFDoA treatment groups (0.2 and 0.5 mg/kg/day) and the control. A positive value signifies up-regulation after treatment; a negative value signifies down-regulation after treatment.

^d^ The indentified proteins were grouped according to their functions.

^e^ Sequence coverage.
