# Supplementary material for: Biological Responses to Perfluorododecanoic Acid Exposure in Rat Kidneys as Determined by Integrated Proteomic and Metabonomic Studies
Source: PLoS One. 2011 Jun 3;6(6):e20862. doi: 10.1371/journal.pone.0020862 (PMC3108999; doi:10.1371/journal.pone.0020862)
Supplement: Table S4 — Fold changes of free amino acids in renal tissue detected by iTRAQ®–LC –MS/MS. (DOCX) [file pone.0020862.s007.docx]

**Table S4** Changes of free amino acid in renal tissue detected by iTRAQ^®^–LC–MS/MS

|  |  |  | | |
| --- | --- | --- | --- | --- |
| **Amino acid** | **Abbreviation** | **Fold change (treated vs control)** | | |
|  |  | **0.05** **mg/kg/d** | **0.2 mg/kg/d** | **0.5 mg/kg/d** |
| Argnine | Arg | 1.13 | 1.14^*^ | 1.10^*^ |
| Histidine | His | 1.22 | 1.24^**^ | 1.24^*^ |
| Isoleucine | Ile | 1.18 | 1.26^*^ | 1.19^**^ |
| Leucine | Leu | 1.20 | 1.24^*^ | 1.23^*^ |
| Lysine | Lys | 0.82 | 0.96 | 0.98 |
| Methionine | Met | 1.25^*^ | 1.30^*^ | 1.24^*^ |
| Phenylalanine | Phe | 1.23 | 1.22^*^ | 1.20^*^ |
| Threonine | Thr | 1.08 | 1.10^*^ | 1.14^*^ |
| Tryptophan | Trp | 1.17 | 1.23^*^ | 1.21^*^ |
| Valine | Val | 1.20 | 1.21^*^ | 1.18^**^ |
| γ-Aminobutyric Acid | GABA | 0.62^**^ | 0.74^*^ | 0.83^**^ |
| Glycine | Gly | 0.96 | 1.05 | 1.03 |
| Serine | Ser | 1.09 | 1.12 | 1.14 |
| Tyrosine | Tyr | 1.32^*^ | 1.32^*^ | 1.32^*^ |
| a-Aminoadipic Acid | Aad | 0.83 | 0.86 | 0.71 |
| Asparagine | Asn | 1.90^*^ | 1.98^*^ | 1.95^*^ |
| Aspartic Acid | Asp | 1.13 | 1.14 | 1.10 |
| Citrulline | Cit | 0.92 | 0.94 | 0.97 |
| Glutamic Acid | Glu | 1.01 | 1.08 | 1.03 |
| Glutamine | Gln | 2.96^*^ | 2.97^*^ | 3.01^*^ |
| Ornithine | Orn | 2.23^*^ | 2.52^**^ | 2.25^**^ |
| Cysteine | Cys | 3.18^*^ | 2.97^**^ | 3.30^**^ |
| Cystathionine | Cth | 1.34 | 0.67^**^ | 0.73^*^ |
| Homocysteine | Hcy | 0.97 | 1.03 | 1.02 |
| a-Aminobutyric Acid | Abu | 0.95 | 1.03 | 1.09 |
| Alanine | Ala | 0.94 | 0.96 | 1.00 |
| Anserine | Ans | 1.65 | 2.13 | 1.62 |
| β-alanine | bAla | 0.78^*^ | 0.93^*^ | 0.87^*^ |
| β-aminoisobutyric acid | bAib | 2.55^*^ | 2.00^*^ | 2.00 |
| Ethionine | EtN | 0.71^*^ | 0.83 | 0.83 |
| δ- Hydroxylysine | Hyl | 0.76^*^ | 0.82 | 0.77 |
| Hydroxyproline | Hyp | 1.01 | 0.90 | 0.92 |
| 1-Methylhistidine | 1MHis | 3.33^*^ | 3.35^**^ | 2.96^*^ |
| 3-Methylhistidine | 3MHis | 1.23^*^ | 1.47^*^ | 1.21^**^ |
| Phosphorylethanolamine | PEtN | 4.06 | 2.62 | 2.87 |
| Phosphoserine | PSer | 1.30 | 1.21^*^ | 1.16^*^ |
| Carnosine | Car | 0.91 | 1.64 | 1.01 |
| Proline | Pro | 1.02 | 1.04 | 1.02^*^ |
| Sarcosine | Sar | 1.06 | 1.21 | 2.05^*^ |
| Arginosuccinic Acid | Asa | 1.10 | 0.94 | 1.14 |
| Homocitrulline | Hcit | 0.73 | 0.53^*^ | 0.46^*^ |
| Norvaline | Nva | 0.88^**^ | 0.90^**^ | 0.90^*^ |
| Norleucine | Nle | 0.92 | 0.91 | 0.91 |

^*^Significant difference from control, *p* < 0.05. ^**^Significant difference from control, *p* < 0.01.
