# Supplementary material for: Biological Responses to Perfluorododecanoic Acid Exposure in Rat Kidneys as Determined by Integrated Proteomic and Metabonomic Studies
Source: PLoS One. 2011 Jun 3;6(6):e20862. doi: 10.1371/journal.pone.0020862 (PMC3108999; doi:10.1371/journal.pone.0020862)
Supplement: Text S1 — Protocols of quantitative PCR and western blot. (DOC) [file pone.0020862.s008.doc]

**Text S1**

**Supplementary Methods**

**Quantitative PCR.** Total RNA was isolated from the liver samples using the TRIzol reagent (Invitrogen Corp., Carlsbad, CA) according to the manufacturer’s instructions. The concentration of total RNA was measured by absorbance at 260 nm using a UV1240 spectrophotometer (Shimadzu, Japan). The purity was estimated by the 260/280 nm absorbance ratio. One microgram of total RNA was subjected to reverse transcription using an oligo-(dT)_15_ primer (Promega, USA) and M-MuLV reverse transcriptase (New England Biolabs, UK) according to the manufacturers’ instructions. PCR primers were designed using Primer Premier 5.0 software. Gene names, accession numbers, forward and reverse primer sequences, and product length are listed in Supplementary Table 1. PCR amplification was conducted on a Stratagene Mx3000P qPCR system (Stratagene, USA). The reaction mixture was composed of 9 μl of SYBP RealMasterMix (Tiangen, Beijing, China), forward and reverse primers (10 μM, 0.5 μl each), 9 μl of nuclease-free water, and the cDNA sample (1 μl). In the 2-D DIGE results, the protein expression levels of beta actin (Actb) and tubulin, which are traditionally used as housekeeping genes, were significantly altered. Therefore, five potential reference genes (Actb, glyceraldehyde-3-phosphate dehydrogenase (Gapd), Hprt, tyrosine 3-monooxygenase (Ywhaz), and hydroxymethylbilane synthase (Hmbs)) were chosen, and their gene expression stability was tested by quantitative PCR and GeNorm v 3.5 software (PrimerDesing Ltd.) analysis [32]. Hprt had the greatest stability in the current study (data not shown) and, thus, was chosen as the reference for the western blot and quantitative PCR analyses. ,. The PCR amplification protocol was 95 ºC for 2 minutes followed by 40 cycles of 94 ºC for 15 seconds, 56 ºC for 15 seconds, and 72 ºC for 15 seconds. After PCR, a melting curve analysis was performed to demonstrate the specificity of the PCR product, which was displayed as a single peak. Every sample was analyzed in triplicate. Differences in expression levels were calculated using the 2^-∆∆Ct^ method[30].

**Western blot.** The independent protein samples extracted for DIGE (six per groups) were used for western blot analysis to validate the results from 2-D-DIGE. The proteins were denatured for 5 min at 95°C, and 50 µg was separated by 12% SDS-PAGE and then transferred onto the polyvinylidene ﬂuoride (PVDF) membrane (GE Healthcare).After blocking by non-fat milk (5%), the PVDF membrane were incubated with primary antibodies including rabbit monoclonal antibodies to human fructose-1,6- biphosphatase 1 (Fbp1, Proteintech Group, Chicago, IL, USA) , human isovaleryl coenzyme A dehydrogenase (Ivd, Proteintech Group, Chicago, IL, USA), rat malate dehydrogenase 1(Mdh1, AVIVA, Beijing, China), rat dihydrolipoamide S-acetyltransferase (Dlat, AVIVA, Beijing, China) , rat Hprt (Santa Cruz, USA), and goat monoclonal antibodies to rat pyruvate carboxylase (Pc, LifeSpan, IN, USA) overnight at 4°C, respectively. Antibody concentrations used were based on the manufacturer’s specifications. After incubation with primary antibodies, membranes were washed and incubated with an appropriate horseradish peroxidase (HRP)-conjugated secondary antibody (goat anti-rabbit IgG or rabbit anti-goat IgG) (Boster Biological Technology, Wuhan, China). Then the protein bands were visualized by enhanced chemiluminescence (superECL, Tigen, Beijing, China). The densitometry analyses were done using a Tianeng Image Analyzer with GIS2008 software. Data were normalized to protein expression levels of Hprt.
